# Factors Affecting Medication Adherence in Patients with Mechanical Heart Valves Taking Warfarin: The Role of Knowledge on Warfarin, Medication Belief, Depression, and Self-Efficacy

**DOI:** 10.3390/ijerph18105214

**Published:** 2021-05-14

**Authors:** Soohyun Park, Insil Jang

**Affiliations:** 1 Department of Nursing, Asan Medical Center, Seoul 05505, Korea; pharaam@amc.seoul.kr; 2 Department of Nursing, Chung-Ang University, Seoul 06974, Korea

**Keywords:** cardio-thoracic nursing, medication management, clinical decision making, health education, depression

## Abstract

Non-adherence is highlighted as one of the main contributors to the occurrence of adverse events and negative clinical outcomes in patients treated with warfarin. The aim was to examine knowledge on warfarin, medication belief, depression, and self-efficacy as factors influencing medication adherence for anticoagulation control. This was a cross-sectional study. The participants in this study were patients who visited an outpatient clinic of cardiovascular surgery to administer anticoagulants after mechanical valve replacement surgery at a tertiary hospital in Seoul. Responses of 154 participants on questionnaires were analyzed from 10 September to 26 December 2020. Multiple regression analyses were performed to assess the factors influencing medication adherence among the patients with anticoagulation control. Factors influencing medication adherence were consuming warfarin for 3 to 5 years, awareness of target prothrombin time international normalized ratio, knowledge of warfarin, and depression. Medication beliefs and self-efficacy had no significant influence on medication adherence. The most important factors associated with medication adherence in patients with mechanical heart valves were knowledge about warfarin and depression. In the control of oral anticoagulants that require continuous management, education and providing accurate guidance is more important than personal preferences. Clinical nurses should facilitate educational programs tailored to the characteristics of the patient, including their purpose and method of taking warfarin, specific diets, their knowledge on warfarin’s interaction with other drugs, symptoms of adverse events, and self-management. In addition, healthcare providers should check whether warfarin therapy is being controlled by evaluating medication adherence and depression levels among patients.

## 1. Introduction

The number of degenerative diseases is increasing due to population aging. Each year, there are more than 300,000 cases of mechanical valve replacement worldwide [1]. In Korea, more than 2500 mechanical valve replacements are performed annually, accounting for 25–30% of adult cardiac surgeries [2]. Mechanical heart valves are more durable than bio-prostheses but are more thrombogenic. Oral anticoagulants, which must be taken for life after mechanical valve replacement, show therapeutic effects by maintaining adequate blood levels with the international normalized ratio (INR) of prothrombin time (PT) depending on the valve location. According to racial and sociocultural characteristics, low-dose anticoagulant therapy is recommended for Asians based on coagulation characteristics, lifestyle (including food), interactions with other drugs, and vulnerable thrombotic complications [2,3]. Oral anticoagulant therapy after mechanical valve replacement in Koreans requires more detailed, systematic, and continuous management [2]. Warfarin intake requires special care as it has a narrow therapeutic concentration range, and its therapeutic effect will reduce if not properly dosed [4]. Despite concerns related to its therapeutic range and multiple drug–food interactions, warfarin is the mainstay of oral anticoagulation in patients with mechanical heart valves. However, continued management could cause stress due to the risk of bleeding and blood clots, which can lead to valve failure, heart failure, reoperation, increased medical expenses, poor quality of life, and death. Therefore, having a plan for correct medication use is essential [5,6]. For life-long management, taking warfarin to maintain valve function is crucial, and individual counselling and management on anticoagulation control is required to maintain therapeutic INR for older patients with various underlying diseases [7].

The social cognition theory [8] and previous studies indicate that a combination of factors should be considered while studying health-related behaviors, such as medication adherence. Physiological aspects, interaction of the medicine with other medication, the intake of food containing vitamin K, patient knowledge on drug therapy, demographic and clinical characteristics, medication belief, and treatment adherence may interfere with oral anticoagulant therapy treatment success [9,10]. According to the World Health Organization, medication adherence is the degree to which a person’s behavior, represented by drug consumption, diet tracking, and lifestyle changes, agrees with a doctor or other health professional’s recommendations [11]. Non-adherence is one of the main contributors to the occurrence of adverse events, such as thromboembolism and bleeding in warfarin users; it can lead to increased morbidity, mortality, and healthcare costs [12]. Therefore, factors specific to warfarin, such as frequent PT-INR monitoring, knowledge about warfarin, recognition of signs of adverse events, and patient behavior towards warfarin therapy, should be considered while assessing medication adherence to warfarin therapy [13,14]. High levels of drug knowledge and self-efficacy increase the level of drug use and self-care. The importance of having warfarin-related drug knowledge increases with age. Patients might find instructions for use complicated and feel burdened because of taking regular medication. Additionally, with the advancement of PT-INR home monitoring, more specific and accurate drug knowledge is required. The main causes of inappropriate drug delivery are lack of information provided by medical personnel and lack of education tailored for each patient; however, consultation with nurses for knowledge improvement helps improve drug delivery [13,14,15]. Self-efficacy is a person’s belief that they can perform the necessary actions to achieve a desirable outcome; higher self-efficacy leads to higher confidence in achieving one’s goals. Self-efficacy connects knowledge and behavior, and one can improve self-efficacy by increasing drug knowledge through appropriate drug-related education, which can in turn promote drug-taking performance [16].

Further, mood may also be related to medication adherence. Depression is common among chronically ill patients. Patients with mechanical valves have to be aware of the therapeutic effects of oral anticoagulants, check for symptoms of adverse events, and pay attention to their medication’s interaction with other drugs and foods. Due to the stress from managing these medication-related factors, they might experience symptoms of depression [12]. Depression decreases patients’ interest in the disease or treatment, negatively affects drug use, and leads to a lack of social support and treatment pessimism [17]. Depression is an independent predictor of medication adherence in patients with chronic diseases including cardiovascular diseases [18]. Additionally, medication belief is also related to medication adherence. Medication belief refers to the patient’s cognition and is classified into the perceived need and concern related to drugs. Treatment beliefs about medication include necessity beliefs, which are related to the perceived need for treatment, and concern beliefs, which are related to side effects, dependence, and tolerance to medication [19]. Medication belief during treatment is a stronger predictor of adherence than other clinical or demographic variables [12,19].

According to previous studies, factors influencing medication adherence are demographic factors, social support (such as family support), knowledge about medication and diseases, depression, self-efficacy, adverse events, discomfort, and duration of drug use. However, there is a dearth of studies that provide comprehensive explanations on the topic. Studies on oral anticoagulants in the past have focused on simple correlations between each variable affecting medication adherence and progression. Thus, the purpose of this study was to explore the following: (a) the knowledge about warfarin, medication belief, depression, self-efficacy, and medication adherence among patients with a mechanical heart valve; (b) the relationship between the knowledge about warfarin, medication belief, depression, self-efficacy, and medication adherence; and (c) the factors influencing medication adherence among patients with anticoagulation control.

## 2. Materials and Methods

### 2.1. Study Design

The study has a cross-sectional design and was conducted in 2020. The study is reported according to the Strengthening of the Reporting of Observational studies [20].

### 2.2. Participants and Data Collection

The participants comprised patients who visited an outpatient cardiac hospital to administer anticoagulants after having a mechanical valve replacement surgery at a general hospital in Seoul. The inclusion criteria were (a) participants who had taken warfarin for more than six months after mechanical valve replacement; (b) participants aged 18 years or older; (c) PT-INR values refer only to the results of hospital tests; and (d) participants who were able to communicate and respond to the questionnaire. We explained the study’s purpose to the prospective participants. Those who agreed to participate were selected. The exclusion criteria for the participants were being over 80 years of age, patients taking warfarin after tissue valve replacement, and refusal to participate in the study. The required sample size was calculated through the G-power 3.1. program. The significance level, effect size, and power were set at α = 0.05, f 2= 0.15 (medium), Power (1 − β) = 0.95, and predictors 5, respectively, based on the results of a linear multiple regression analysis, which showed the required sample size was 138.

Questionnaires were distributed to 170 patients, to account for a possible attrition rate of 20%. All questionnaires were distributed from 10 September 2020 to 26 December 2020, of which 160 were filled and returned (return rate: 94.1%). After excluding the incomplete questionnaires, data from 154 participants were used in the final analyses. Therefore, the sample size was appropriate. After obtaining approval from the institutional review board, the coordinator directly distributed and collected the questionnaires among participants who visited the cardiac hospital. After receiving written consent from the participants, their responses were collected in an envelope. The self-report questionnaire took about 20 to 30 min to fill, after which a reciprocal gift was provided.

### 2.3. Ethical Considerations

This study was approved by the institutional review board (IRB No. 2020-1323) of the investigator’s affiliate hospital. All procedures were performed in accordance with the Declaration of Helsinki (1964) and its later amendments or comparable ethical standards. Informed consent was obtained from all participants included in the study.

### 2.4. Data Collection Tools

The knowledge of anticoagulant therapy was measured using the anticoagulant drug knowledge tool developed by Jang [21]. It comprises 13 items: the purpose of taking medication (1), medication administration (3), diet (2), concomitant medications (4), and occurrence and management of side effects (3). Correct responses were scored as 1, and wrong answers or ‘I don’t know’ were scored as 0. A higher total score indicated a higher degree of drug knowledge. During the development of the tool, Cronbach’s ⍺ was 0.63. In this study, Cronbach’s ⍺ was 0.70.

The BMQ-specific (Beliefs about Medicines Questionnaire-specific) developed by Horne et al. [22] was translated into Korean by Kim and Min [23]. We used the Korean version of the BMQ-specific to measure participants’ belief in medication. Its validity and reliability were verified. It comprises 10 questions: necessity of medication (5) and medication concerns (5). Responses for each question are marked on a 5-point Likert scale ranging from 1 = ‘not at all’ to 5 = ‘very much’, and the range of the summation score for each sub-area is 5–25 points. A higher score indicates that medication is needed and the participant is concerned about medication [22]. The medication belief score is calculated by subtracting the concern score from the necessity score. The medication belief score ranges from −20 to 20, and the larger the positive value, the more positive the level of belief, and the larger the negative value, the more negative the belief. Regarding reliability, the original tool’s Cronbach’s α was 0.83, and the Cronbach’s α of the Korean version of the BMQ-specific was 0.80. In this study, Cronbach’s ⍺ was 0.74.

A self-report depression scale was developed by Radloff (affiliated with the National Institute of Mental Health) [24]. Chon et al. [25] developed an integrated Korean version of the scale called the CES-D (Center for Epidemiologic Studies Depression Scale). It comprises 20 questions, and the depressive symptoms experienced over the past week are measured on a 4-point Likert scale (0: extremely rare, 1 day, or less; 1: occasional, 1–2 days in a week; 2: often, 3–4 days; 3: most days, 5 days, or more). The total score ranges from 0–60, and the higher the total score, the severe the depression. The positive questions are summed using inverse transformation. During the development of the tool, Cronbach’s α was 0.85, and the Cronbach’s α of the scale developed by Chon et al. [25] was 0.91. In this study, Cronbach’s ⍺ was 0.89.

Self-efficacy was measured using the Chronic Disease Self-Efficacy Scale-Korean version (CDSES-K), a tool standardized in Korean by Kim et al. [26], which is based on the Chronic Disease Self-Efficacy Scale (CDSES) developed by Lorig et al. [27]. It comprises 32 questions covering 8 areas, including the following items: controlling symptom management (7) and controlling depression (5). We included the following questions: regular exercise (3); illness-related information (1); help received through family, friends, or community (4); communicating with medical staff (3); general disease management (5); and housework (4). Each question is measured on a 10-point scale, and a higher score indicates higher self-efficacy. During the development of the tool, the Cronbach’s α was 0.96. In this study, Cronbach’s ⍺ was 0.94.

The Morisky Medication Adherence Scale (MMAS-8) is a self-report tool developed by Morisky et al. [28]. To measure medication adherence, we used the Korean version of the scale, translated by Min and Kim [16]. Excluding the fifth inverse question, questions 1–7 are measured with 0 points for ‘yes’ and 1 point for ‘no’. For question 8, 1 point is given for ‘0 = almost never’, 0.75 points for ‘1 = very occasionally’, 0.5 points for ‘2 = sometimes’, and 0.5 points for ‘3 = sometimes’. It is measured as 0.25 points and 0 points for ‘4 = always yes’. The maximum score is 8. A score less than 6 points indicates low progression, a score of 6 points or more and less than 8 points indicates medium progression, and 8 points indicates high transition. During tool development, Cronbach’s α was 0.83, and in Min and Kim’s study, Cronbach’s α was 0.72. In this study, Cronbach’s ⍺ was 0.73.

The following demographic characteristics were assessed: gender, age, marital status, household status, education level, current occupation, economic status, PT-INR, operation name, additional reason for warfarin therapy except for valve replacement, duration of warfarin consumption, awareness of target INR, warfarin-related adverse events, and education or counselling for anticoagulant control.

### 2.5. Data Analyses

Data were analyzed using SPSS version 25.0 software (SPSS Inc.). Before the analyses, the data were examined for outliers, missing responses, and incomplete responses. We calculated the mean and standard deviations of participants’ scores on knowledge about warfarin, medication belief, depression, self-efficacy, and medication adherence. Multicollinearity was confirmed by calculating tolerance (<0.10) and the variance inflation factor (<2). The normality of standardized residuals (Shapiro–Wilk test), homoscedasticity (Durbin–Watson test), and hypothesis of independence (plot) were also confirmed. In addition to demographic characteristics, a warfarin consumption period of 3–5 years and having information about target PT-INR were converted into dummy variables. Correlations between scores on knowledge about warfarin, medication belief, depression, self-efficacy, and medication adherence were assessed using Pearson’s correlation. A multiple regression was performed to assess the factors influencing medication adherence among the patients with anticoagulation control.

## 3. Results

### 3.1. Demographic and Clinical Characteristics of Participants

Of the 154 participants, 51.3% were male, 85.1% were married, 89.6% lived with their family, 61.7% were currently working, and 64.3% had been taking warfarin for more than 5 years. The mean age was 54.05 ± 10.78, and the mean of PT-INR was 2.28 ± 0.49. Of the total number of participants, 40.3% underwent the surgery with aortic valve replacement, 38.3% with mitral valve replacement, and 9.7% had experienced a warfarin-related adverse event (Table 1).

### 3.2. Knowledge on Warfarin, Medication Belief, Depression, Self-Efficacy, and Medication Adherence

The mean knowledge on warfarin score for all patients was 10.02 ± 2.05. The mean of medication belief was 3.27 ± 4.77: necessity was 16.23 ± 4.19 and concerns was 12.96 ± 3.66. The mean scores for depression, self-efficacy, and medication adherence were 8.82 ± 9.27, 7.25 ± 1.66, and 3.20 ± 1.30, respectively (Table 2).

Regarding medication adherence based on the demographic and clinical characteristics of the patients, main differences were observed on the basis of the administration period for warfarin, awareness of target INR, and experiencing a warfarin-related adverse event (Table 3).

### 3.3. Correlation between Knowledge on Warfarin, Medication Belief, Depression, Self-Efficacy, and Medication Adherence

Medication adherence exhibited a negative correlation with depression (r = −0.223, *p* = 0.006), and a positive correlation with knowledge on warfarin (r = 0.290, *p* < 0.001), medication belief (r = 0.220, *p* = 0.006), and self-efficacy (r = 0.184, *p* = 0.022). No significant correlations were found between medication belief, depression, and self-efficacy (Table 4).

### 3.4. Factors Influencing Medication Adherence

Factors positively influencing medication adherence were warfarin consumption of 3–5 years (β = 0.246, *p* = 0.001) and knowledge on warfarin (β = 0.195, *p* = 0.015). On the other hand, factors negatively influencing medication adherence were having information on target PT-INR (β = −0.244, *p* = 0.009) and depression (β = −0.238, *p* = 0.005). The explanatory power of the model was statistically significant (19.9%, F = 12.756, *p* < 0.001) (Table 5).

## 4. Discussion

We now discuss our results in the context of chronically ill patients taking warfarin due to the lack of prior research on the factors affecting medication adherence among heart valve surgery patients taking warfarin. In this study, similar to previous studies, the knowledge related to warfarin was high [15,29]. Patients (77.3%) who had consumed warfarin for more than 3 years had increased knowledge about warfarin due to long-term use. Medication belief and self-efficacy scores were also similar to previous studies. However, as compared to those with other chronic diseases, for this study’s participants, medication adherence and depression were low [12,13,14,30]. In the case of patients with chronic diseases, administration of drugs has a practical effect on symptom reduction, but a direct connection between warfarin administration and symptoms is not immediately confirmed. As educational contents for warfarin management, nurses should emphasize the importance of taking medication and its mechanism and insist on the importance of medication adherence.

Patients who consumed warfarin for 3–5 years and those who experienced warfarin-related adverse events showed high medication adherence. Similar to previous studies, we found that the longer the drug administration period, the higher the patients’ knowledge about medication [12,15]. Additionally, patients who experienced warfarin-related adverse events showed high interest in self-management due to high medication adherence, similar to previous research. However, patients who did not know the therapeutic target INR for valve function accurately had higher medication adherence than those who did. Rather than knowing only the target INR, specific and detailed knowledge about the purpose of drug administration, the management of adverse events, interaction with diet and other medication, and counselling focused on specific situations is necessary [4]. Experiencing events, such as bleeding due to warfarin, a concomitant disease, or a drug interaction that increases the anticoagulant effect, might have a negative effect on follow-up management and contribute to the patient’s stress. Further, patients taking warfarin constantly face difficulties in improving their health and eating habits; therefore, it is necessary to provide information on these topics through an educational intervention. In clinical practice, nurses should strive to develop programs involving face-to-face health education-related activities to ensure that the educational intervention is personalized according to the patients’ level of knowledge.

Factors related to medication adherence were the duration of administration, target INR, knowledge related to warfarin, and depression; however, medication belief and self-efficacy did not contribute to continuing medication use. Target INR perception had a negative effect on medication adherence; therefore, simply remembering INR levels might not be enough. Regular INR testing and clinical appointments for warfarin may help allay concerns and negative attitudes towards warfarin [4,15]. Anxiety about adverse events and unclear information about warfarin could be addressed by highlighting the need for support and provision of information by healthcare professionals [12,20].

Understanding patients’ behavior is the first step toward improving self-management behavior including medication adherence. In our study, medication beliefs did not influence medication adherence. As several drugs are prescribed to patients with heart disease, it is difficult to identify the therapeutic mechanism of each drug and assess the benefit of each treatment [14,15,29]. Nevertheless, if one’s beliefs related to warfarin are negative, they may affect medication choice and adherence; further studies could explore this. Among the variables influencing medicine administration, self-efficacy is an important behavioral change factor. Among the variables influencing drug administration, self-efficacy is an important behavioral change factor, and Bandura said that when self-efficacy is controlled, there is no predictive power for the behavior of expecting results [8]. However, heart disease is directly related to life, and it is interpreted that the importance and severity of disease management are relative to the more experienced and actively coping compared to those with other diseases. Heart disease patients might be aware of the severity of their condition and actively cope with it. Additionally, self-efficacy can vary with age, and the higher one’s age, the lower their overall self-efficacy, due to functional decline [15]. Future research could explore the relationship between the changes in self-efficacy with age and medication adherence as well as the associations between medication belief, self-efficacy, and medication adherence using path analysis or structural equation model.

Educational experience influences medication-related knowledge, leading to active participation by the patient due to increased interest in the medication process. The structure of medication-related education can positively change patients’ attitudes towards medication intake and improve their quality of life by recognizing negative experiences with drugs as manageable [4,15]. Accurate medication-related knowledge is crucial for increasing medication adherence in patients taking warfarin. By raising their awareness about the disease and medication through educational experiences, patients can take control of their treatment. Patients can actively participate in self-management by increasing medication adherence.

Finally, we found that depression influenced medication adherence, and the higher the degree of depression, the lower the medication adherence. This is consistent with the results of previous studies [17,18,30]. The depression-related scores of patients with mechanical heart valves were lower than that of patients with other chronic conditions. The depression of valvular replacement patients is lower than that of existing chronic patients, but this may be because they are the participants of continuous outpatient visits that are not related to social isolation, absence of medical staff, or financial difficulties. Depression directly or indirectly affects medication adherence through self-efficacy and perceived side effects [12,30]. Therefore, in life-long management, using a depression measurement tool during the basic assessment to assess the patient’s mental health condition is essential. This will help gauge the need for psychological counselling to be included in the intervention program. Further, it is necessary to check whether the effectiveness of medication adherence can be improved by increasing the patients’ ability to cope with and manage their own problems by implementing self-management programs, which can increase their ability to cope with diseases and their self-efficacy.

This study has several limitations as follows. First, the patients were recruited from only one hospital, which limits the generalizability of our results. There is a need for replication studies that include patients with mechanical heart valves from other hospitals and communities. Moreover, as we aimed to explore relationships and potential correlates of medication adherence, caution is required when interpreting differences between medication groups. Second, our findings were based on self-report and self-administered surveys, which might have led to biased responses. In future research, electronic methods could be used in conjunction with self-report measures to triangulate the results and improve the reliability of the medication adherence assessment. Third, the cross-sectional nature of the study suggests that causation was not implied. Consequently, authors of further longitudinal research could examine whether medication belief and self-efficacy predict adherence to oral anticoagulants.

## 5. Conclusions

Factors including frequent INR monitoring, knowledge about warfarin, recognition of signs of adverse events, and other factors including the nature of behaviors should be considered while assessing patients’ adherence to warfarin therapy. This study examined knowledge about warfarin, medication belief, depression, and self-efficacy as factors influencing medication adherence. Medication belief and self-efficacy were not found to be correlated with medication adherence related to warfarin. Clinician nurses should promote medication adherence by emphasizing the importance of taking medications and the mechanisms of medications as part of patient education for warfarin control. Significant factors associated with adherence in patients with mechanical heart valves were related to having specific knowledge on warfarin and depression. Thus, nurse specialists and nutritionists should provide educational programs tailored to the characteristics of the patient, including the purpose and method of taking warfarin, specific dietary requirements, information on warfarin’s interaction with other drugs, symptoms of adverse events, and self-monitoring strategies for self-management. In particular, healthcare providers should monitor warfarin therapy by evaluating medication adherence and facilitate increased adherence by assessing patients’ depression levels. The results of this study suggest a direction toward educational content to increase medication adherence through non-face-to-face methods used in patient education, intervention, and counseling after the COVID-19 situation.

## Figures and Tables

**Table 1:** General characteristics and clinical details of participants (n = 154).

Factors		Categories	n (%)	Mean ± SD
General factors	Gender	Male Females	79(51.3) 75(48.7)	
	Age (in years)	<45 45–54 55–64 ≥65	26(16.9) 49(31.8) 49(31.8) 30(19.5)	54.05 ± 10.78
Socio-environmental factors	Marital status	Single Married Others	18(11.7) 131(85.1) 5(3.2)	
	Household	With family Live alone Others	138(89.6) 13(8.5) 3(1.9)	
	Education	Middle school or less High school College or more	23(14.9) 58(37.7) 73(47.4)	
	Employment status	Yes No	95(61.7) 59(38.3)	
	Economic status	High Middle Low	18(11.7) 106(68.8) 30(19.5)	
Clinical factors	PT-INR			2.28 ± 0.49
	Operation name	MVR AVR DVR (MVR + AVR) TVR PVR	59(38.3) 62(40.3) 28(18.2) 2(1.3) 3(1.9)	
	Additional reason for warfarin therapy except valve replacement	CVA Atrial fibrillation CVA & atrial fibrillation Others	24(15.6) 49(31.8) 10(6.5) 4(2.6)	
	Years of warfarin consumption	≤1 <1–≤3 <3–≤5 >5	6(3.9) 29(18.8) 20(13.0) 99(64.3)	
	Having information on target PT-INR	Yes No	118(76.6) 36(23.4)	
	Warfarin-related adverse event	Yes No	15(9.7) 139(90.3)	
	Education or counseling about warfarin control	Yes No	82(53.2) 72(46.8)	

Abbreviations: PT-INR = prothrombin time international normalized ratio; MVR = mitral valve replacement; AVR = aortic valve replacement; DVR = double valve replacement; TVR = tricuspid valve replacement; PVR = pulmonary valve replacement; CVA = cerebrovascular accident.

**Table 2:** Knowledge on warfarin, medication belief, depression, self-efficacy, and medication adherence (n = 154).

Variables	Min	Max	Mean (SD)	Range
Knowledge on warfarin	3.00	13.00	10.02 (2.05)	0–13
Medication belief	−7.00	16.00	3.27 (4.77)	−20–20
Necessity	5.00	25.00	16.23 (4.19)	5–25
Concerns	5.00	25.00	12.96 (3.66)	5–25
Depression	0.00	49.00	8.82 (9.27)	0–60
Self-efficacy	1.94	10.00	7.25 (1.66)	1–10
Medication adherence	0.75	8.00	3.20 (1.30)	0–8

**Table 3:** Medication adherence according to participants’ demographic and clinical characteristics (n = 154).

Variables		n (%)	Medication Adherence	
			Mean (SE)	F or *t (p)* Duncan
Gender	Male Females	79 (51.3) 75 (48.7)	3.31 (0.16) 3.09 (0.14)	1.074 (0.302)
Age (in years)	<45 45–54 55–64 ≥65	26 (16.9) 49 (31.8) 49 (31.8) 30 (19.5)	3.11 (0.24) 3.28 (0.17) 3.11 (0.19) 3.29 (0.28)	0.238 (0.870)
Marital status	Single Married Others	18 (11.7) 131 (85.1) 5 (3.2)	3.10 (0.28) 3.17 (0.11) 4.40 (0.96)	2.262 (0.108)
Household	With family Live alone Others	138 (89.6) 13 (8.5) 3 (1.9)	3.09 (0.10) 4.10 (0.48) 4.33 (1.30)	4.953 (0.058)
Education	Middle school or less High school College or more	23 (14.9) 58 (37.7) 73 (47.4)	3.32 (0.55) 3.24 (0.17) 3.13 (0.13)	0.726 (0.538)
Current occupation	Yes No	95 (61.7) 59 (38.3)	3.22 (0.13) 3.17 (0.17)	0.057 (0.812)
Economic status	High Middle Low	18 (11.7) 106 (68.8) 30 (19.5)	2.83 (0.30) 3.16 (0.12) 3.56 (0.29)	1.925 (0.149)
Operation name	MVR ^a^ AVR ^b^ DVR (MVR + AVR) ^c^ TVR ^d^ PVR ^e^	59 (38.3) 62 (40.3) 28 (18.2) 2 (1.3) 3 (1.9)	3.09 (0.17) 3.19 (0.15) 3.26 (0.23) 6.63 (0.12) 2.83 (0.60)	3.941 (0.005) d > a,b,c,e
Additional reason for warfarin therapy except valve replacement	CVA Atrial fibrillation CVA and atrial fibrillation Others	24 (15.6) 49 (31.8) 10 (6.5) 4 (2.6)	3.23 (0.27) 3.01 (0.18) 2.75 (0.37) 4.44 (0.26)	1.630 (0.170)
Years on warfarin	≤1 ^a^ <1–≤3 ^b^ <3–≤5 ^c^ >5 ^d^	6 (3.9) 29 (18.8) 20 (13.0) 99 (64.3)	2.67 (0.47) 2.90 (0.22) 4.06 (0.31) 3.15 (0.13)	4.089 (0.008) c > a,b,d
Having information on target PT INR	Yes No	118 (76.6) 36 (23.4)	3.00 (0.09) 3.86 (0.28)	13.08 (<0.001)
Warfarin-related adverse event	Yes No	15 (9.7) 139 (90.3)	4.02 (0.50) 3.11 (0.10)	6.797 (0.010)
Education or counseling about warfarin control	Yes No	82 (53.2) 72 (46.8)	3.21 (0.15) 3.19 (0.15)	0.004 (0.951)

Abbreviations: PT-INR = prothrombin time international normalized ratio; MVR = mitral valve replacement; AVR = aortic valve replacement; DVR = double valve replacement; TVR = tricuspid valve replacement; PVR = pulmonary valve replacement; CVA = cerebrovascular accident.

**Table 4:** Correlations matrix indicating relationships between knowledge on warfarin, medication belief, depression, self-efficacy, and medication adherence (n = 154).

Variables	r (*p*)				
	Knowledge on Warfarin	Medication Belief	Depression	Self-Efficacy	Medication Adherence
Knowledge on warfarin	1				
Medication belief	0.234 (0.003)	1			
Depression	−0.208 (0.010)	−0.073 (0.371)	1		
Self-efficacy	0.273 (0.001)	0.136 (0.092)	−0.454(<0.001)	1	
Medication adherence	0.290 (<0.001)	0.220 (0.006)	−0.223 (0.006)	0.184 (0.022)	1

**Table 5:** Multiple regression analysis to identify the factors influencing medication adherence (n = 154).

Variable	B	β	t	*p*
Constant Duration of warfarin consumption (<3–≤5)	4.641 0.946	0.246	6.170 3.344	<0.001 0.001
Having information on target PT-INR	−0.832	−0.244	−3.184	0.009
Knowledge on warfarin Depression	0.123 −0.052	0.195 −0.238	2.469 −2.804	0.015 0.005

F = 12.756, *p* < 0.001, R^2^ = 0.220, Adj. R^2^ = 0.199.
